# Fully Physically Crosslinked Hydrogel with Ultrastretchability, Transparency, and Freezing-Tolerant Properties for Strain Sensor

**DOI:** 10.3390/ma17205102

**Published:** 2024-10-18

**Authors:** Pengbo Shang, Yang Ji, Feng Ji

**Affiliations:** 1 The Department of Panel Factory, Xiamen Tianma Display Technology Co., Ltd., Xiamen 361101, China; pengboshang@163.com; 2 College of Chemical Engineering and Materials Science, Quanzhou Normal University, Quanzhou 362000, China

**Keywords:** conductive hydrogel, stretchability, anti-freezing, strain sensor

## Abstract

Nowadays, conductive hydrogels show significant prospects as strain sensors due to their good stretchability and signal transduction abilities. However, traditional hydrogels possess poor anti-freezing performance at low temperatures owing to the large number of water molecules, which limits their application scope. To date, constructing a hydrogel-based sensor with balanced stretchability, conductivity, transparency, and anti-freezing properties via simple methods has proven challenging. Here, a fully physically crosslinked poly(hydroxyethyl acrylamide)–glycerol–sodium chloride (PHEAA–Gl–NaCl) hydrogel was obtained by polymerizing hydroxyethyl acrylamide in deionized water and then soaking it in a saturated NaCl solution of glycerol and water. The PHEAA–Gl–NaCl hydrogel had good transparency (~93%), stretchability (~1300%), and fracture stress (~287 kPa). Owing to the presence of glycerol and sodium chloride, the PHEAA–Gl–NaCl hydrogel had good anti-freezing properties and conductivity. Furthermore, the PHEAA–Gl–NaCl hydrogel-based strain sensor possessed good sensitivity and cyclic stability, enabling the detection of different human motions stably and in a wide temperature range. Based on the above characteristics, the PHEAA–Gl–NaCl hydrogel has broad application prospects in flexible electronic materials.

## 1. Introduction

Nowadays, flexible strain sensors, which can convert mechanical deformations into detectable electronic signals, are attracting growing attention in the fields of human motion detection, intelligent robotics, and electronic skins [1,2,3]. These applications require strain sensors to be stretchable and conductive. Normally, elastomers and hydrogels are widely used to design strain sensors [4,5]. Elastomer-based strain sensors are often composed of elastomer substrates (e.g., polyurethane, polydimethylsiloxane, silicone rubber, etc.) and conductive nanofillers (e.g., carbon nanotubes, metal nanoparticles, MXene nanosheets, graphene, etc.) [6,7,8]. For example, Yang et al. engineered a multifunctional sensor based on carboxylic styrene butadiene rubber and sodium-polyacrylate-modified MXene, which could accurately detect body movements at various scales [9]. However, owing to the poor stretchability and greater hardness than soft human skin, their practical use scope is limited [10]. Meanwhile, as the conductive nanofillers are opaque, the transparency of these sensors is always undesirable, which will limit their application in visualization [11].

Compared with elastomer-based strain sensors, hydrogel-based sensors, with the advantage of good skin-like properties, have aroused greater interest in the field of wearable sensors [12,13]. The hydrogels used in the sensor should have good flexibility, conductivity, and transparency [14,15]. Good flexibility ensures that the hydrogel can withstand repeated mechanical bending deformation [16]. Conductivity enables the hydrogel to convert mechanical deformation signals into electrical signals [17]. Transparency ensures that the hydrogel can be used in a visual environment [18].

Generally, there are two ways to endow hydrogels with good conductivity. The first approach is to introduce carbon-based materials or conductive polymers (e.g., carbon nanotubes, MXene, graphene, polypyrrole, polythiophene, etc.) into the hydrogel [19,20]. For example, Patel et al. synthesized a multifunctional hydrogel using carboxymethyl chitosan and unzipped carbon nanotubes via a one-pot strategy; the hydrogel-based strain sensor could detect human motions [21]. However, the application of such hydrogels was limited owing to their inherent black color. Moreover, the conductive components in the hydrogel could easily agglomerate, which led to low mechanical properties and compromised conductivity [22]. The other approach is to introduce inorganic salt ions (e.g., NaCl, LiCl, KCl, etc.) into the hydrogel [23,24]. For instance, Zheng et al. constructed a polyacrylamide–carrageenan hydrogel containing binary cations Zn^2+^ and Li^+^. The binary cations endowed the hydrogel with excellent ionic conductivity [25]. Among the conductive fillers, inorganic salts have been primarily considered because they not only have the lowest cost but also endow hydrogels with ionic conductivity and transparency [26]. Conventional hydrogels inevitably freeze at low temperatures, which severely inhibits their use in low-temperature environments. Thus, in addition to the properties mentioned above, anti-freezing properties are also necessary for hydrogels [27]. Glycerol, as a nontoxic, viscous liquid, is often used to prepare anti-freezing hydrogels because it can form hydrogen bonds with water molecules and decrease the freezing point of water molecules [28,29].

Hydrogels can be divided into physically crosslinked hydrogels and chemically crosslinked hydrogels according to whether chemical crosslinkers are added in the preparation process. Compared with chemically crosslinked hydrogels, physically crosslinked hydrogels are more widely considered by researchers due to their relatively simple preparation process. Hydroxyethyl acrylamide (HEAA) is a monomer containing two hydrogen bond donors in the form of free hydroxyl and amide groups [30]. HEAA can be polymerized in water and glycerol media [31]. Meanwhile, hydrogen bonds among PHEAA molecules can form PHEAA networks, even in the absence of chemical crosslinkers [32]. Therefore, as a new gel material, HEAA has been widely considered. For example, Liu et al. prepared a PHEAA-based hydrogel by incorporating tannic acid–MXene into a PHEAA network in a glycerol/water binary solvent, and the obtained hydrogel exhibited good anti-freezing properties. However, the color of the hydrogel was black owing to the color of MXene [33]. Our group also carried out a large number of studies based on PHEAA-based hydrogels. In our previous work, we synthesized physically crosslinked poly(hydroxyethyl acrylamide)–gelatin–glycerol–lithium chloride (PHEAA–GE–Gl–LiCl), poly(hydroxyethyl acrylamide)–ethylene glycol–sodium chloride (PHEAA–GE–EG–NaCl), and poly(hydroxyethyl acrylamide)–glycerol–lithium chloride (PHEAA–Gl–LiCl) hydrogels by a one-pot method [34,35,36]. In these systems, the hydrogels possessed conductivity and anti-freezing properties due to the presence of inorganic salts and cryoprotectants. However, in the process of preparing these gels using the one-pot method, we found that the high content of salts and cryoprotectants weakened the hydrogen bonds in the hydrogels. Therefore, the content of salts and cryoprotectants in these systems was limited. As a result, the conductivity and anti-freezing properties were not satisfactory [34,35,36]. Meanwhile, the PHEAA–GE–Gl–LiCl and PHEAA–GE–EG–NaCl hydrogels were non-transparent owing to the existence of gelatin [34,35]. Although great advances have been made in PHEAA-based hydrogel sensors, preparing a PHEAA hydrogel-based sensor with good transparency, conductivity, and anti-freezing properties is still a major challenge.

In this work, a fully physically crosslinked poly(hydroxyethyl acrylamide)–glycerol–sodium chloride (PHEAA–Gl–NaCl) hydrogel was prepared by polymerizing hydroxyethyl acrylamide in deionized water and then soaking it in a saturated NaCl solution of glycerol and water. It was expected that the PHEAA–Gl–NaCl hydrogel would have stretchability and transparency properties. Meanwhile, the PHEAA–Gl–NaCl hydrogel should have good anti-freezing and conductivity properties owing to the large amount of glycerol and sodium chloride. Thus, the PHEAA–Gl–NaCl hydrogel could detect various human motions in a wide temperature range. This research provides a new idea for the preparation of strain sensors with balanced stretchability, conductivity, transparency, and anti-freezing properties.

## 2. Materials and Methods

### 2.1. Materials

Hydroxyethyl acrylamide (HEAA), glycerol (Gl), sodium chloride (NaCl), and Irgacure 2959 (UV initiator) were purchased from Shanghai Aladdin Reagent Co., Ltd. (Shanghai, China).

### 2.2. Fabrication of PHEAA–Gl–NaCl Hydrogels

The PHEAA–Gl–NaCl hydrogels were prepared as follows. First, a mixture of HEAA (40 wt%), Irgacure 2959 (1 mol% of HEAA), and deionized water was stirred at 60 °C for 1 h. Subsequently, the homogeneous solution was exposed to UV radiation for 3 h, which led to the polymerization of a HEAA monomer to obtain a PHEAA hydrogel. Next, the PHEAA hydrogel was soaked in a saturated NaCl solution of glycerol/water (1:1, *w*/*w*) for 2 h to produce the PHEAA–Gl–NaCl hydrogel. Furthermore, for comparison, a series of hydrogels were prepared by varying the immersion time and the weight ratio of glycerol to water.

### 2.3. Characterization

The chemical structures of the hydrogels were obtained by a Thermo Scientific Nicolet iS50 FTIR spectrometer (Waltham, MA, USA) in the range of 4000–700 cm^−1^ in the ATR mode. The transmittance of the hydrogel was measured by a UV–vis spectrometer (PE LAMBDA 365, Los Angeles, CA, USA). The storage modulus (G′) and loss modulus (G″) at 25 °C were tested on a rheometer (DHR-2, TA Instruments, New Castle, DE, USA) with a parallel plate geometry. The strain amplitude sweep (0.01–1%) was conducted at 1 Hz. The freezing points of the hydrogels were characterized by differential scanning calorimetry (DSC 25, TA Instruments, USA). The DSC measurements were performed from 20 °C to −70 °C at 2 °C/min. The swelling ratio (SR) was measured by soaking the PHEAA hydrogel in different media. At defined time intervals, the hydrogels were removed and weighed. The SR was calculated as follows: $SR={(W}_{t}-W_{0})/W_{0}\times 100\%$, where *W_t_* is the weight of the swollen hydrogel and *W_0_* is the weight of the initial PHEAA hydrogel.

### 2.4. Mechanical Test

In the tensile test, the gel samples were cut into dumbbell shapes. Their mechanical properties were tested by a tensile machine (Instron 3369, Norwood, MA, USA). The fracture stress (σ), elongation at break (ε), and elastic modulus (E) were obtained according to our previous report [36].

### 2.5. Electrical Test

The conductivity of the hydrogels (σ, S/m) was measured by an electrochemical workstation according to the equation *σ = L*/(*R × A*), where *L* denotes the length between the connecting negative and positive terminals, and *R* and A denote the resistance and the cross-sectional area of the hydrogels, respectively.

### 2.6. Application of PHEAA–Gl–NaCl Hydrogel-Based Sensor

The sensing performance for imitated human motions was obtained by manually bending the joint components of the wooden mode. The PHEAA–Gl–NaCl hydrogel-based strain sensor was placed at different human joints, and the electrical signals were measured by the electrochemical workstation. To evaluate the sensing performance of the sensor at −40 °C, the sensor was adhered to the prosthetic hand/wooden human model and kept at −40 °C. The sensing performance was assessed by bending the joints manually.

## 3. Results

### 3.1. Preparation of PHEAA–Gl–NaCl Hydrogel

**Figure 1:**
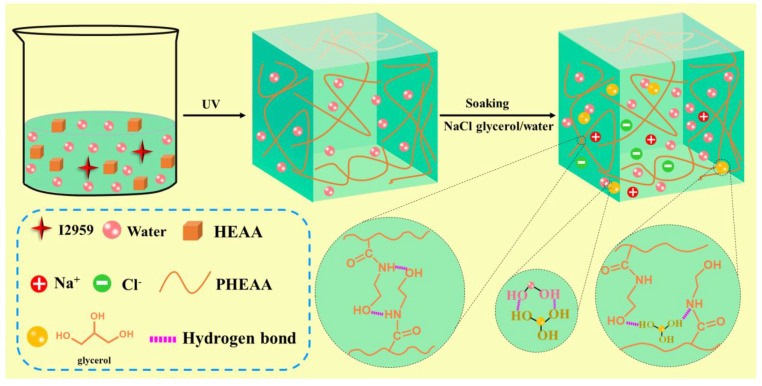
Preparation procedure of PHEAA–Gl–NaCl hydrogel.

Figure 1 shows the preparation process of the PHEAA–Gl–NaCl hydrogel. First, Irgacure 2959 and HEAA were added to deionized water and the mixture was heated to 60 °C for 1 h to form a homogeneous solution. Then, the above solution was exposed to UV light for 3 h to initiate the polymerization of a HEAA monomer to obtain a PHEAA hydrogel. In the PHEAA hydrogel, the gel network was formed via hydrogen bonding among the PHEAA chains [36]. Subsequently, the PHEAA hydrogel was soaked in a saturated NaCl solution of glycerol/water (1:1, *w*/*w*) for 2 h to produce the PHEAA–Gl–NaCl hydrogel. In the preparation process of the PHEAA–Gl–NaCl hydrogel, no chemical crosslinkers were added, so the PHEAA–Gl–NaCl hydrogel was fully physically crosslinked.

**Figure 2:**
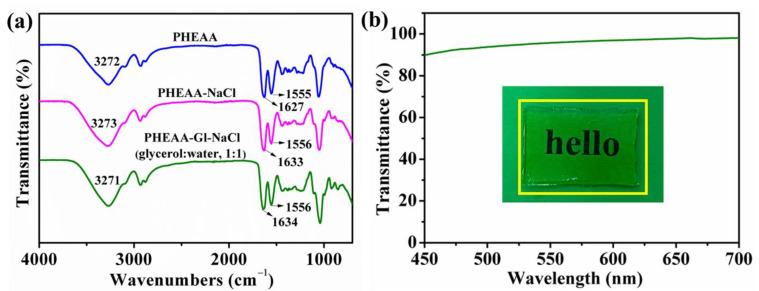
(**a**) FTIR spectroscopy of the hydrogels. (**b**) Transparency of the PHEAA–Gl–NaCl hydrogel.

The structure of the PHEAA–Gl–NaCl hydrogel was analyzed via FT-IR spectroscopy (Figure 2a). In the spectrum of the PHEAA hydrogel, the band at 3272 cm^−1^ belonged to O–H and N–H stretching vibrations. The peak at 1627 cm^−1^ belonged to C=O stretching vibration, and the peak at 1555 cm^−1^ belonged to N–H bending vibration [36]. In the spectrum of the PHEAA–NaCl hydrogel, the band at 3273 cm^−1^ belonged to the stretching vibration of O−H and N−H, and the peak at 1633 cm^−1^ belonged to the stretching vibration of C=O. Compared with the PHEAA–NaCl hydrogel, a slight shift was observed in the stretching vibration of O−H and N−H (from 3273 cm−1 to 3271 cm−1) and C=O (from 1633 cm−1 to 1634 cm−1). Compared with the PHEAA hydrogel, no new absorption band appeared in the PHEAA–NaCl and PHEAA–Gl–NaCl hydrogels, confirming that no chemical reactions occurred. Moreover, the PHEAA–Gl–NaCl hydrogel had excellent transparency, and the word “hello” under the PHEAA–Gl–NaCl hydrogel could be clearly observed by the naked eye. The transmittance value was around 93% at a 500 nm wavelength (Figure 2b).

### 3.2. Mechanical Properties of PHEAA–Gl–NaCl Hydrogel

**Figure 3:**
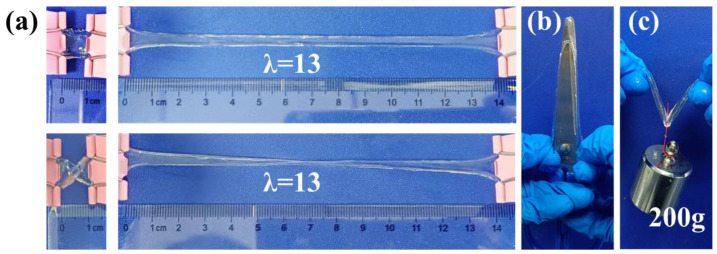
(**a**) Photos of PHEAA–Gl–NaCl hydrogel (glycerol/water, 1:1) in states of (**a**) stretching, (**b**) puncture resistance, and (**c**) withstanding a load of 200 g.

The mechanical properties of the PHEAA–Gl–NaCl hydrogel were explored to determine its applicability as a flexible device. As shown in Figure 3, the PHEAA–Gl–NaCl hydrogel could be easily stretched to 13 times its original length. It did not break, even in a distorted state. Meanwhile, the PHEAA–Gl–NaCl hydrogel had good toughness to resist the puncture and was able to withstand a load of 200 g. The above result preliminarily confirmed the good mechanical properties of the PHEAA–Gl–NaCl hydrogel.

**Figure 4:**
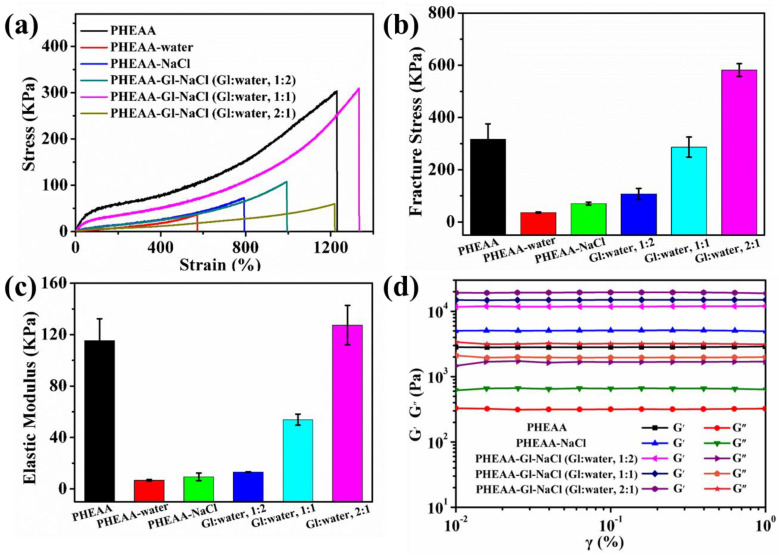
(**a**) Stress–strain curves, (**b**) fracture stress, (**c**) elastic moduli, and (**d**) storage and loss moduli of the hydrogels.

The soaking time and the type of immersion medium had an important effect on the mechanical properties. Thus, we systematically explored their effects on the stress–strain of the hydrogels. As shown in Figure 4a–c, the fracture stress, elastic modulus, and elongation at break of the original PHEAA hydrogel were 320 KPa, 87.49 KPa, and 1381%, respectively. When the PHEAA hydrogel was immersed in different media for 30 min, the mechanical properties of the resulting hydrogel decreased sharply (Figures S1 and S2). The fracture stress of the PHEAA–water, PHEAA–NaCl, PHEAA–Gl–NaCl (Gl–water, 1:2), PHEAA–Gl–NaCl (Gl–water, 1:1), and PHEAA–Gl–NaCl (Gl–water, 2:1) hydrogels was 53 KPa, 92 KPa, 150 KPa, 181 KPa, and 302 KPa, respectively. This was because the hydrogel rapidly absorbed a large amount of the soaking medium at the initial stage of soaking and decreased the crosslinking density of the hydrogel sharply (Figure S3). As shown in Figure S3, the swelling ratios of the PHEAA–water, PHEAA–NaCl, PHEAA–Gl–NaCl (Gl–water, 1:2), PHEAA–Gl–NaCl (Gl–water, 1:1), and PHEAA–Gl–NaCl (Gl–water, 2:1) hydrogels at 30 min were 129%, 66%, 41%, 29%, and 15%, respectively. Upon continuing to increase the immersion time to 3 h, the mechanical curves of the hydrogels obtained in the different media showed different trends. The fracture stress of the PHEAA–water, PHEAA–NaCl, and PHEAA–Gl–NaCl (Gl–water, 1:2) hydrogels continued to decrease to 21 KPa, 50 KPa, and 68 KPa, respectively. The fracture stress of the PHEAA–Gl–NaCl (Gl–water, 1:1) and PHEAA–Gl–NaCl (Gl–water, 2:1) hydrogels first decreased and then increased, and it reached the maximum value at 2 h. Therefore, we chose the hydrogel obtained by soaking for 2 h for the subsequent experiment. Under the immersion time of 2 h, the fracture stress of the PHEAA–water, PHEAA–NaCl, PHEAA–Gl–NaCl (Gl–water, 1:2), PHEAA–Gl–NaCl (Gl–water, 1:1), and PHEAA–Gl–NaCl (Gl–water, 2:1) gels was 36 KPa, 70 KPa, 107 KPa, 287 KPa, and 582 KPa, respectively (Figure 4b,c). Meanwhile, we found that all of the PHEAA–Gl–NaCl hydrogels possessed good elongation at break (≥983%) (Figure S4).

In addition, the elastic moduli of the hydrogels were also calculated. As shown in Figure 4c, the elastic moduli of the PHEAA–water, PHEAA–NaCl, PHEAA–Gl–NaCl (Gl–water, 1:2), PHEAA–Gl–NaCl (Gl–water, 1:1), and PHEAA–Gl–NaCl (Gl–water, 2:1) hydrogels were 7 KPa, 9 KPa, 13 KPa, 54 KPa, and 127 KPa, respectively. It was easy to find that the elastic moduli of these hydrogels increased in turn, indicating that the crosslink density of these hydrogels also increased sequentially [36]. The photographs of these hydrogels are shown in Figure S5. As shown in Figure S5, after soaking the PHEAA hydrogel in different media for 2 h, the size of the obtained hydrogels was in the order of PHEAA–water, PHEAA–NaCl, PHEAA–Gl–NaCl (Gl–water, 1:2), PHEAA–Gl–NaCl (Gl–water, 1:1), and PHEAA–Gl–NaCl (Gl–water, 2:1) from large to small. This result further implied that the crosslink density of these hydrogels increased sequentially in this order.

Moreover, strain–sweep was also carried out to study the rheological behavior of the hydrogels. As shown in Figure 4d, all of the hydrogels displayed strain-dependent viscoelastic behavior. In the linear viscoelasticity region (0.01–1%), the storage modulus (G′) was always greater than the loss modulus (G″), indicating that the hydrogels had elastic, solid-like behavior [37,38]. Meanwhile, the G′ of the PHEAA–Gl–NaCl hydrogel (Gl–water, 2:1) was the largest, followed by the PHEAA–Gl–NaCl (Gl–water, 1:1), PHEAA–Gl–NaCl (Gl–water, 1:2), PHEAA–NaCl, and PHEAA gels. The results also confirmed that the crosslink density of the PHEAA–Gl–NaCl hydrogel (Gl–water, 2:1) was the largest, followed by the PHEAA–Gl–NaCl (Gl–water, 1:1), PHEAA–Gl–NaCl (Gl–water, 1:2), PHEAA–NaCl, and PHEAA–water hydrogels.

### 3.3. Anti-Freezing Properties of PHEAA–Gl–NaCl Hydrogel

**Figure 5:**
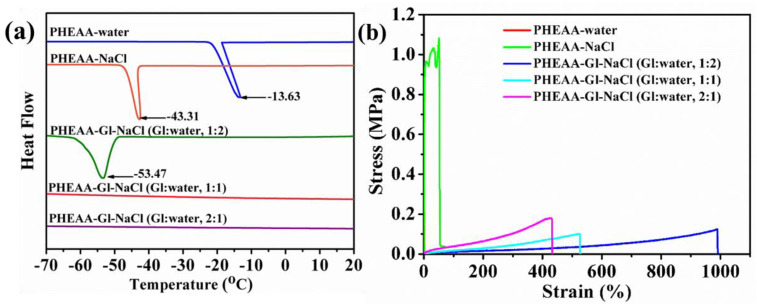
(**a**) DSC and (**b**) tensile curves at −40 °C for different hydrogels.

The water molecules in a hydrogel at a low temperature will inevitably freeze through hydrogen bonds, resulting in the loss of its elasticity and conductivity. Therefore, anti-freezing properties are very important for hydrogels [39]. In this work, in order to endow the hydrogels with anti-freezing properties, sodium chloride and glycerol were diffused into them. DSC was used to characterize the freezing points of the hydrogels. As displayed in Figure 5a, the freezing point of the PHEAA–water hydrogel was −13.63 °C. Compared with the PHEAA–water hydrogel, the freezing point of the PHEAA–NaCl hydrogel decreased to −43.31 °C owing to the colligative property of diffused sodium and chloride ions [40]. Further, the freezing point of the PHEAA–Gl–NaCl hydrogel (Gl–water, 1:2) dramatically decreased to −53.47 °C. Surprisingly, the exothermic peaks of the PHEAA–Gl–NaCl (Gl–water, 1:2) and PHEAA–Gl–NaCl (Gl–water, 2:1) hydrogels entirely disappeared. These results suggested that the diffused glycerol could decrease the freezing point effectively by forming hydrogen bonds with the water molecules [41]. At the same time, to further confirm this result, the mechanical properties of the hydrogels at −40 °C were investigated, and their stress–strain curves are shown in Figure 5b and Figure S6. Compared with that at 25 °C, the elongation at break of the PHEAA–water and PHEAA–NaCl hydrogels at −40 °C was only 1.39% and 52.81%, respectively. These results suggested that the water molecules in the hydrogels froze at −40 °C. In contrast, all of the PHEAA–Gl–NaCl hydrogels still possessed higher fracture strain (433–989%). This result was caused by the existing glycerol in the PHEAA–Gl–NaCl hydrogel, which hindered the crystallization of water and improved the anti-freezing ability.

**Figure 6:**
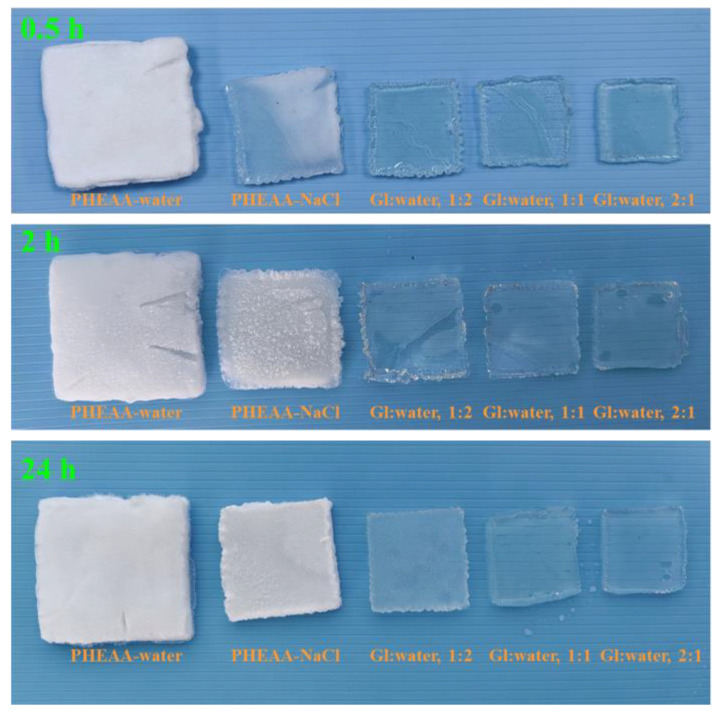
Photographs of different hydrogels at −40 °C at different times.

Moreover, photographs of the hydrogels at −40 °C are shown in Figure 6. Compared with the original transparent state (Figure S5), the PHEAA–water hydrogel become completely white after 0.5 h at −40 °C, indicating that the water in the hydrogel had frozen. Compared with the PHEAA–water hydrogel, the PHEAA–NaCl hydrogel only showed partial whitening for 0.5 h, indicating that sodium chloride gave the PHEAA–NaCl hydrogel an anti-freezing property to a certain extent. Compared with the PHEAA–water and PHEAA–NaCl hydrogels, all of the PHEAA–Gl–NaCl hydrogels were transparent at −40 °C for 24 h, indicating that the PHEAA–Gl–NaCl hydrogels had excellent anti-freezing properties because of the presence of glycerol. In our previous work, a PHEAA–Gl–LiCl hydrogel was prepared by the one-pot method. However, owing to the limited, relatively low glycerol content, the PHEAA–Gl–LiCl hydrogel could only resist freezing for 2 h at −40 °C [36]. Compared with the PHEAA–Gl–LiCl hydrogel, the PHEAA–Gl–NaCl hydrogel obtained by the immersion method possessed clearly better anti-freezing abilities.

### 3.4. Conductivity of PHEAA–Gl–NaCl Hydrogel

**Figure 7:**
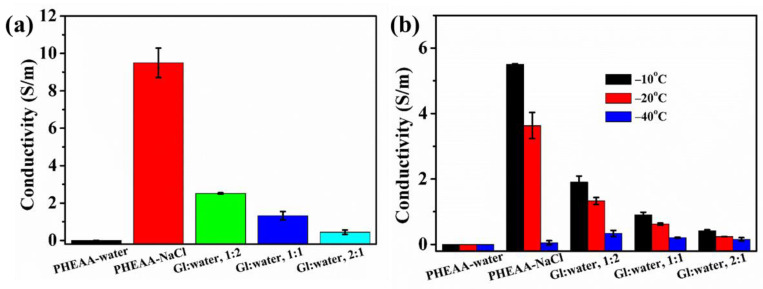
(**a**) The conductivity of the hydrogels at room temperature. (**b**) The effect of the temperature on the conductivity of the hydrogels.

Hydrogels used as strain sensors should have good conductivity [42]. In this study, a conductive PHEAA–Gl–NaCl hydrogel was prepared by soaking the PHEAA hydrogel in a saturated NaCl solution of glycerol and water. As shown in Figure 7a, due to the absence of conductive ions, the conductivity of the PHEAA–water hydrogel was zero. Compared with the PHEAA–water hydrogel, the conductivity of the PHEAA–NaCl hydrogel rapidly increased to 9.502 S/m, which suggested that a large number of sodium and chloride ions was successfully diffused into the hydrogel. The conductivities of the PHEAA–Gl–NaCl (Gl–water, 1:2), PHEAA–Gl–NaCl (Gl–water, 1:1), and PHEAA–Gl–NaCl (Gl–water, 1:1) hydrogels were 2.516 S/m, 1.320 S/m, and 0.443 S/m, respectively. It was easy to find that the higher the glycerol content in the immersion medium, the lower the conductivity of the obtained hydrogel. This was because the higher the glycerol content in the immersion medium, the less sodium chloride in the immersion medium was dissolved, and thus the number of ions diffused into the hydrogel was also reduced.

For a hydrogel-based sensor to be used at low temperatures, it should also have excellent conductivity at low temperatures. Therefore, we also studied the conductivity of the hydrogels at low temperatures in detail. As shown in Figure 7b, when the temperature decreased from room temperature to a low temperature, the conductivities of all hydrogels decreased, because the movement of the conductive ions was more difficult at a low temperature. At −40 °C, the conductivities of the PHEAA–NaCl, PHEAA–Gl–NaCl (Gl–water,1:2), PHEAA–Gl–NaCl (Gl–water, 1:1), and PHEAA–Gl–NaCl (Gl–water, 2:1) hydrogels were 0.048 S/m, 0.338 S/m, 0.207 S/m, and 0.154 S/m, respectively. Although the conductivity of the PHEAA–NaCl hydrogel was the highest at room temperature, its conductivity at −40 °C was the lowest. This phenomenon was because the water molecules in the PHEAA–NaCl hydrogel partially or completely froze (Figure 6). The above results again indicate that glycerol played a significant role in providing the anti-freezing property among the hydrogels. Furthermore, the conductivity of the PHEAA–Gl–NaCl hydrogel prepared in this work and the other PHEAA-based hydrogels prepared in our previous work were compared. As shown in Table S1, the PHEAA–Gl–NaCl hydrogel had the highest electrical conductivity because it contained more conductive ions [34,35,36,43].

### 3.5. Performance as Strain Sensor

**Figure 8:**
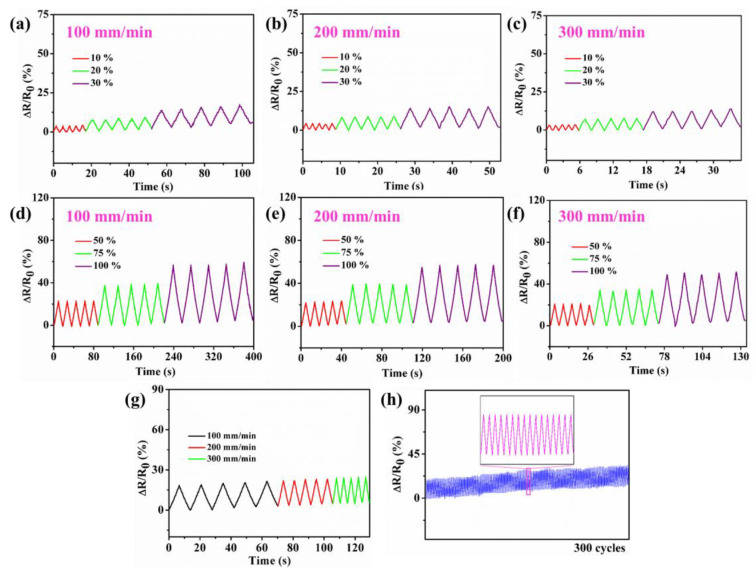
Relative resistance variations of PHEAA–Gl–NaCl hydrogel (**a**–**f**) at different rates and strains, (**g**) at different rates under cyclic 40% tensile strain, and (**h**) under cyclic 40% tensile strain for 300 cycles.

Figure 8a−g show that the PHEAA–Gl–NaCl hydrogel-based strain sensor outputted stable and repeatable signals at different strains (10–100%) and rates (100 mm/min, 200 mm/min, 300 mm/min). Figure 8h shows that the strain sensor could also output repeatable signals over 300 cycles at 40% tensile strain, which indicates that the PHEAA–Gl–NaCl hydrogel possessed good stability.

**Figure 9:**
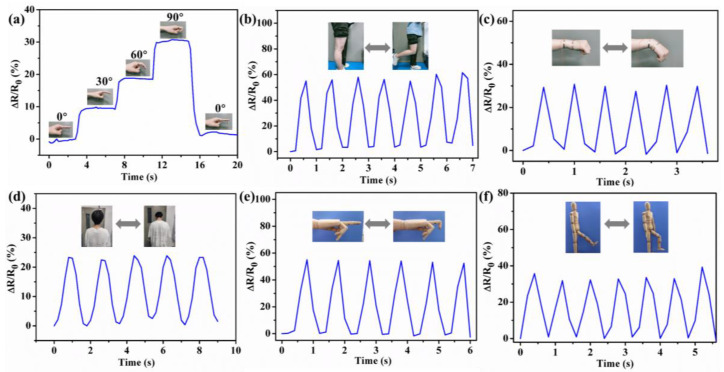
Relative resistance changes induced by bending (**a**) finger, (**b**) knee, (**c**) wrist, and (**d**) neck at room temperature. Relative resistance changes induced by bending (**e**) finger of prosthetic hand and (**f**) knee of wooden human model at −40 °C.

Based on its good ultrastretchability, conductivity, transparency, and sensing performance, the PHEAA–Gl–NaCl hydrogel was designed as a strain sensor to detect different human movements. Figure 9a–d show the relative resistance changes of the strain sensor when monitoring joint bending (e.g., finger, knee, wrist, and neck). It was found that the relative resistance increased gradually during joint bending. Once the joint bending was released, the relative resistance also decreased to the original value. Meanwhile, because different joints had different deformations, the curves for the bending of different joints were also different. In addition to detecting joint bending at room temperature, the strain sensor could also be applied to detect joint movements at low temperatures. As shown in Figure 9e,f, the strain sensor could detect the bending of a prosthetic hand and the knee of a wooden human model at −40 °C, suggesting that the PHEAA–Gl–NaCl hydrogel-based strain sensor has potential applications in low-temperature environments.

## 4. Conclusions

In conclusion, a fully physically crosslinked PHEAA–Gl–NaCl hydrogel was successfully prepared by polymerizing hydroxyethyl acrylamide in deionized water and then soaking it in a saturated NaCl solution of glycerol and water. The primary driving force for the formation of the PHEAA–Gl–NaCl hydrogel was hydrogen bonds. The PHEAA–Gl–NaCl hydrogel displayed good transparency (∼93%), stretchability (∼1300% elongation), and fracture stress (∼287 kPa). The PHEAA–Gl–NaCl hydrogel also had good anti-freezing (−40 °C) and conductivity (1.32 S/m) properties, owing to the presence of glycerol and sodium chloride. These favorable characteristics meant that the PHEAA–Gl–NaCl hydrogel-based strain sensor possessed good sensitivity and cyclic stability and could monitor various human motions at room and low temperatures. Thus, the PHEAA–Gl–NaCl hydrogel has broad application prospects in flexible electronic materials.

## Data Availability

The raw data supporting the conclusions of this article will be made available by the authors on request.

## Supplementary Materials

The following supporting information can be downloaded at: https://www.mdpi.com/article/10.3390/ma17205102/s1, Figure S1: Stress–strain curves of PHEAA–water, PHEAA–NaCl, PHEAA–Gl–NaCl (Gl–water, 1:2), PHEAA–Gl–NaCl (Gl–water, 1:1), and PHEAA–Gl–NaCl (Gl–water, 2:1) gels obtained by soaking PHEAA hydrogel in different soaking media for different times; Figure S2: The fracture stress of PHEAA–water, PHEAA–NaCl, PHEAA–Gl–NaCl (Gl–water, 1:2), PHEAA–Gl–NaCl (Gl–water, 1:1), and PHEAA–Gl–NaCl (Gl–water, 2:1) gels obtained by soaking PHEAA hydrogel in different soaking media for different times; Figure S3: The swelling ratio (%) of PHEAA–water, PHEAA–NaCl, PHEAA–Gl–NaCl (Gl–water, 1:2), PHEAA–Gl–NaCl (Gl–water, 1:1), and PHEAA–Gl–NaCl (Gl–water, 2:1) gels obtained by soaking PHEAA hydrogel in different soaking media for 3 h; Figure S4: The elongation at break of PHEAA–water, PHEAA–NaCl, PHEAA–Gl–NaCl (Gl–water, 1:2), PHEAA–Gl–NaCl (Gl–water, 1:1), and PHEAA–Gl–NaCl (Gl–water, 2:1) gels obtained by soaking PHEAA hydrogel in different soaking media for different times; Figure S5: Photographs of the original PHEAA hydrogel and the PHEAA–water, PHEAA–NaCl, PHEAA–Gl–NaCl (Gl–water, 1:2), PHEAA–Gl–NaCl (Gl–water, 1:1), and PHEAA–Gl–NaCl (Gl–water, 2:1) gels obtained by soaking PHEAA hydrogel in different soaking media for 2 h; Figure S6: The tensile curves of the PHEAA–water hydrogel at −40 °C; Table S1: Comparison results of the conductivity of the PHEAA–Gl–NaCl hydrogel at room temperature with other PHEAA-based hydrogels obtained in our previous work.
