# Supplementary material for: Fully Physically Crosslinked Hydrogel with Ultrastretchability, Transparency, and Freezing-Tolerant Properties for Strain Sensor
Source: Materials (Basel). 2024 Oct 18;17(20):5102. doi: 10.3390/ma17205102 (PMC11509641; doi:10.3390/ma17205102)
Supplement: Supplementary file 1 [file materials-17-05102-s001.zip › materials-3222324-supplementary.pdf]

Supplementary Materials

1. Figures

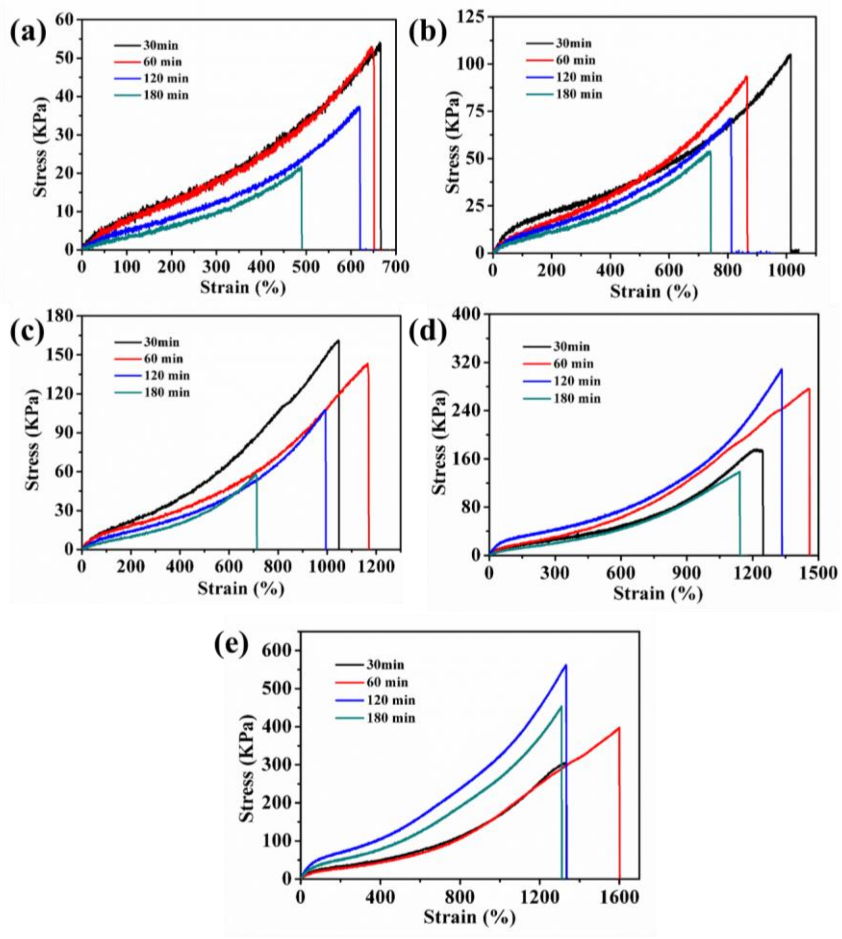

Figure S1. Stress-strain curves of PHEAA-water, PHEAA-NaCl, PHEAA-Gl-NaCl (Gl:water, 1:2), PHEAA-Gl-NaCl (Gl:water, 1:1), and PHEAA-Gl-NaCl (Gl:water, 2:1) gel obtained by soaking PHEAA hydrogel in different soaking media for different time.

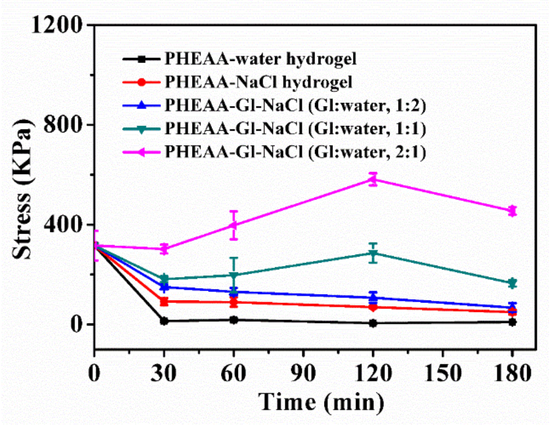

Figure S2. The fracture stress of PHEAA-water, PHEAA-NaCl, PHEAA-Gl-NaCl (Gl:water, 1:2), PHEAA-Gl-NaCl (Gl:water, 1:1), and PHEAA-Gl-NaCl (Gl:water, 2:1) gel obtained by soaking PHEAA hydrogel in different soaking media for different time.

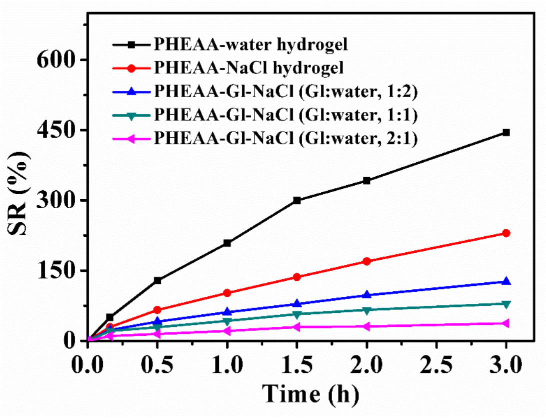

Figure S3. The swelling ratio (%) of PHEAA-water, PHEAA-NaCl, PHEAA-Gl-NaCl (Gl:water, 1:2), PHEAA-Gl-NaCl (Gl:water, 1:1), and PHEAA-Gl-NaCl (Gl:water, 2:1) gel obtained by soaking PHEAA hydrogel in different soaking media for 3 h.

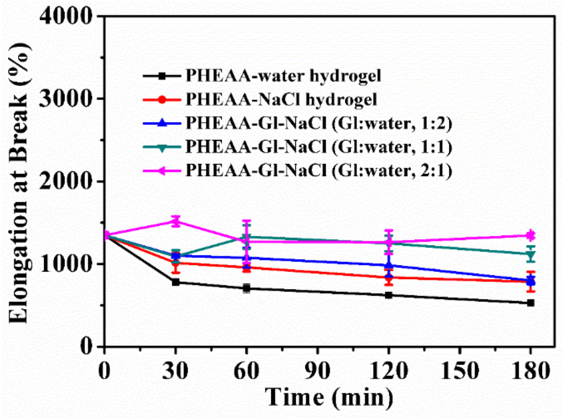

Figure S4. The elongation at break of PHEAA-water, PHEAA-NaCl, PHEAA-Gl-NaCl (Gl:water, 1:2), PHEAA-Gl-NaCl (Gl:water, 1:1), and PHEAA-Gl-NaCl (Gl:water, 2:1) gel obtained by soaking PHEAA hydrogel in different soaking media for different time.

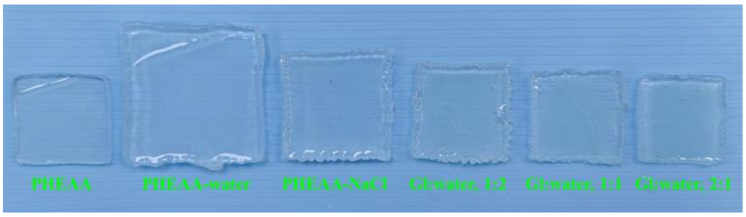

Figure S5. Photographs of the original PHEAA hydrogel, and PHEAA-water, PHEAA-NaCl, PHEAA-Gl-NaCl (Gl:water, 1:2), PHEAA-Gl-NaCl (Gl:water, 1:1), and PHEAA-Gl-NaCl (Gl:water, 2:1) gel obtained by soaking PHEAA hydrogel in different soaking media for 2 h.

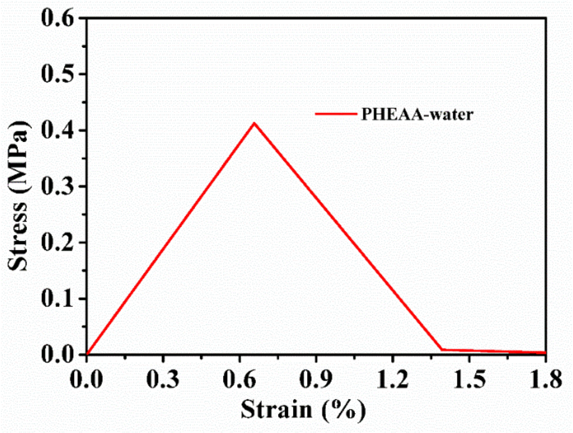

Figure S6. The tensile curves of PHEAA-water hydrogel at −40 °C.

2. Table

Table S1. Comparison results of conductivity of PHEAA-Gl-NaCl hydrogel at room temperature with other PHEAA-based hydrogels in our previous work.

Composition	Conductivity (S/m)	Ref.
**PHEAA-Gl-NaCl**	1.32	This work
PHEAA-GE-Gl-LiCl	0.57	[34]
PHEAA-GE-EG-NaCl	0.53	[35]
PHEAA-Gl-LiCl	0.30	[36]
PHEAA-Car	0.23	[43]
